# Epidemiologic study of ankle fractures in a tertiary hospital

**DOI:** 10.1590/1413-78522014220200874

**Published:** 2014

**Authors:** Marcos Hideyo Sakaki, Bruno Akio Rodrigues Matsumura, Thiago De Angelis Guerra Dotta, Pedro Augusto Pontin, Alexandre Leme Godoy dos Santos, Tulio Diniz Fernandes

**Affiliations:** USP, FM, Hospital das Clínicas, São Paulo, SP, Brazil, Institute of Orthopedics and Traumatology, Hospital das Clínicas, FMUSP, São Paulo, SP, Brazil

**Keywords:** Fractures, bone, Ankle, Epidemiology

## Abstract

**OBJECTIVES:**

To evaluate the epidemiology of ankle fractures surgically treated at the Instituto de Ortopedia e Traumatologia do Hospital das Clínicas da Universidade de São Paulo.

**METHODS:**

Medical records of patients admitted with foot and ankle fractures between 2006 and 2011 were revised. Seventy three ankle fractures that underwent surgical treatment were identified. The parameters analyzed included age, gender, injured side, AO and Gustilo & Anderson classification, associated injuries, exposure, need to urgent treatment, time to definitive treatment and early post-operative complications. Study design: retrospective epidemiological study.

**RESULTS:**

Male gender was predominant among subjects and the mean age was 27.5 years old. Thirty nine fractures resulted from traffic accidents and type B fracture according to AO classification was the most common. Twenty one were open fractures and 22 patients had associated injuries. The average time to definitive treatment was 6.5 days. Early post-operative complications were found in 21.3% of patients.

**CONCLUSIONS:**

Ankle fractures treated in a tertiary hospital of a large city in Brazil affect young people victims of high-energy accidents and present significant rates of associated injuries and post-operative complications.*** Level of Evidence IV, Cases Series.***

## INTRODUCTION

Sports injuries are a major cause of fractures of the foot and ankle, but high-energy trauma are actually responsible for the most serious sequelae. Despite technological evolution of the automobiles, which provides the numerous systems to protect drivers' and passengers' life, lesions on the feet and legs did not decrease in frequency and severity, the deformation of the carrier floor is the factor responsible for trauma in lower limbs.^1^ Fractures of the ankle and foot has significant negative effect on the quality of life of patients, leading to functional disability for numerous activities and often associated with pain.^2^

In polytraumatized patients, fractures and dislocations in ankles and feet are among the most frequently undiagnosed lesions in acute phase.^3^

Fractures of the tibial tarsal joint are among the most treated bone lesions by orthopedic surgeons. Recent observational studies show a significant increase of these lesions between the years 1970 and 2000. In U.S. these fractures are diagnosed in 8.3 cases of 1000 medical visits.^4^

The Brazilian medical literature has few studies related to ankle fractures. Some studies have correlated sports with the type of fracture found; however, the affected population and the characteristics of malleolar fractures have not been described.

Among the 236 patients who sought treatment in the physiotherapy department at a sports club in Minas Gerais because of injuries due to the practice of indoor soccer, 20.1% had injuries in the ankle region, being ankle sprain the more common injury.^5^ Another study conducted over the 23 games of the XV Brazilian Indoor Soccer Championship with teams formed by athletes under 20 years old ("Sub20") conducted in 2004 revealed that among the 32 injuries occurred, the ankle was affected in 18.7% of them, and again the sprain was the most common injury.^6^ In both studies, fractures had no significant effect .

In sailing practice, the most common injury of the foot and ankle region was the lateral sprain, found in 22 of 165 patients practitioners of this sport. Fractures were less frequent, found in four yachtsmen, two with toe fractures, one with calcaneal fracture and one had tibia fracture.^7^

In a study conducted with 930 surf practitioners in Brazil, 13 cases of fracture and 42 sprains in the lower limbs were found, with no specification of the region of the foot and ankle. From a total of 55 cases, the low incidence of 5.9% characterized as rare traumatic injuries in surfers' feet.^8^

In judo, only 6% of injuries are fractures, and despite the foot and ankle are often affected regions, they are overcome by the shoulder, knee and hand.^9^

Debieux *et al*.^10^ studied 387 patients who underwent motorcycle accidents in São Paulo from January 2001 to July 2002, and found that 16% had foot fractures and 12.7% ankle fracture. Of fractures, the most frequent was in the foot region, and only 10% of the entire studied population used boots as protective equipment for the legs. 

Other studies found in the national literature that refer to malleolar fractures are focused on the type of treatment and on the outcomes of surgical treatment.

Baptista *et al*.,^11^ by means of a retrospective study, evaluated the clinical and radiographic results of 70 patients surgically treated for malleolar fractures resulting from different trauma mechanisms from January 1989 to December 1993. They found 80% satisfaction among the patients about the surgery results, but the mean follow-up was short, only two and a half years.

Santin *et al*.^12^ studied the clinical and radiographic results obtained with surgical treatment of 35 patients with Danis-Weber type B malleolar fractures and found good results in 82.8% of them. The surgeries were performed between May 1992 and May 1998, and patients were analyzed retrospectively.

In a prospective study with 21 patients with Danis-Weber type B malleolar fractures operated between March 1999 and July 2001, Tucci Neto *et al.* ^13^ concluded that the use of the fibula plate positioned in the posterolateral region is a good method of treatment when considering aggression to soft tissue and stability of the osteosynthesis.

Schwartsmann *et al.* ^14^ obtained 100% of good results according to the clinical criteria of the American Orthopaedic Foot and Ankle Society in non-surgical treatment of 50 patients with Danis-Weber type B malleolar fractures. The mean follow-up was 48 months, and the inclusion criteria were patients with type B fractures without deviation or satisfactory closed reduction.

The literature review supports the conclusion that there is a shortage in national publications regarding the epidemiology of ankle fractures.

This work aims to evaluate the epidemiological data of ankle fractures treated surgically at our University.

## METHODS

Medical records from every hospitalized patients with fractures of the foot and ankle between 2006 and 2011 at our institution were reviewed. After detailed analysis of these records, 73 cases of surgically treated malleolar fractures were identified.

The parameters evaluated were: age, gender, laterality, injury mechanism, classification (AO and Gustilo & Anderson), associated injuries, exposure, treatment in the ER, time to definitive treatment, early postoperative complications.

This is a retrospective observational epidemiological study based on data survey from medical records of the Institute of Orthopedics and Traumatology, *Hospital das Clinicas, Faculdade de Medicina da Universidade de São Paulo*, SP, Brazil.

## RESULTS

**Figure 1:**
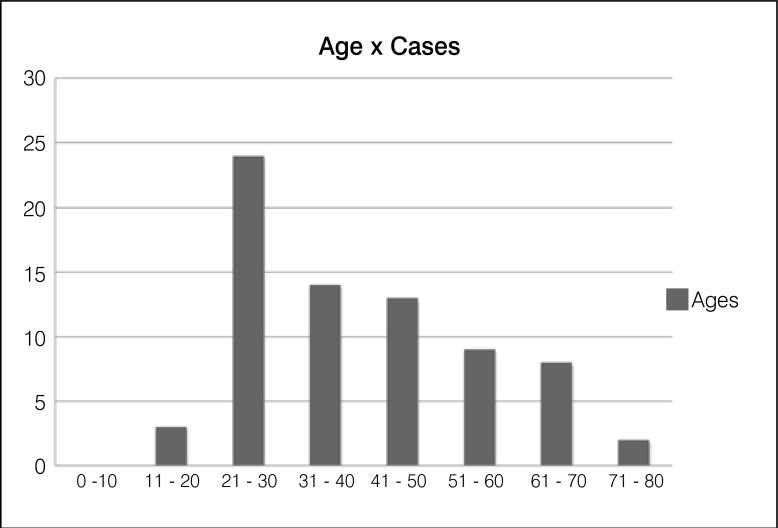
Distribution of cases by age groups.

A total of 73 patients, being 46 males and 27 females, gender ratio of 1.7:1, were evaluated. The age of patients ranged from 17 to 80 years, on average 27.5 years old. (Figure 1) The right hand side was affected in 33 patients and the left in 40.

**Figure 2:**
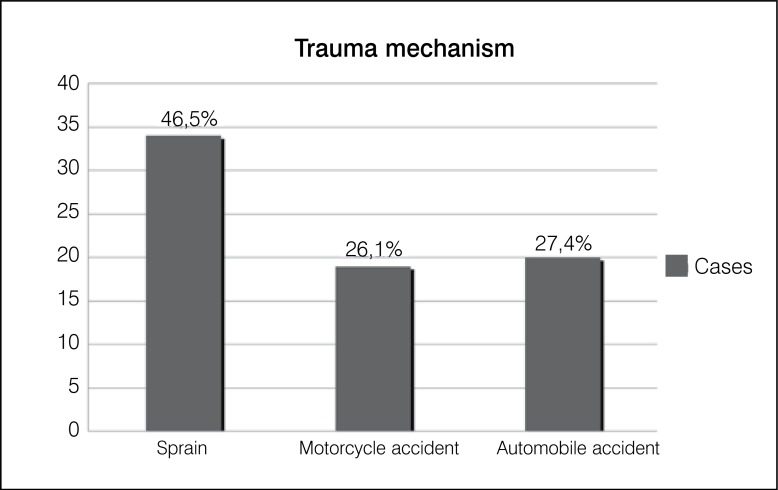
Distribution of cases by trauma mechanism.

The most frequent trauma mechanism was torsional trauma in 34 cases, followed by automobile accidents, with 20 cases, and motorcycle accidents with 19 cases. (Figure 2) Regarding the injury mechanism, 16 cases were in polytraumatized patients.

**Figure 3:**
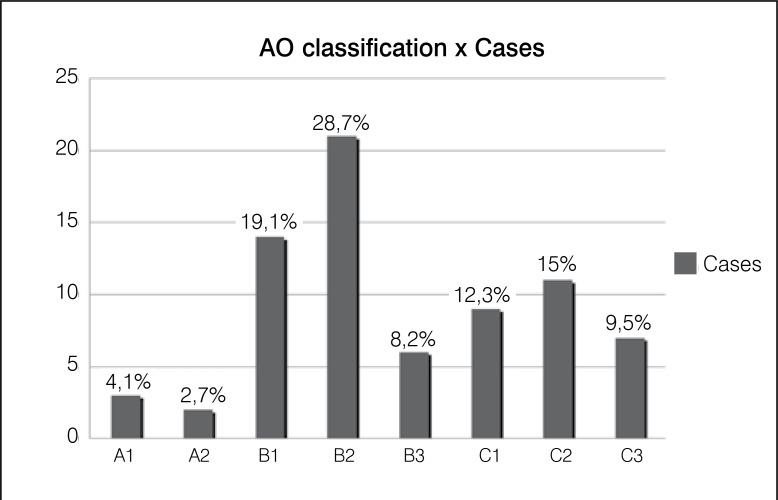
Distribution of cases by AO classification of fractures.

When analyzed according to the AO classification, the most common was B type, with 41 cases, followed by C type with 27 cases and the A type, with five cases. The most common subtype was B2 with 21 cases, representing 28% of treated cases. The distribution of cases according to subtypes is shown in Figure 3.

Twenty-one were compound fractures (28.0%), eight type I, four type II, four type IIIA and five type IIIB, according to the Gustilo and Anderson classification.

**Figure 4:**
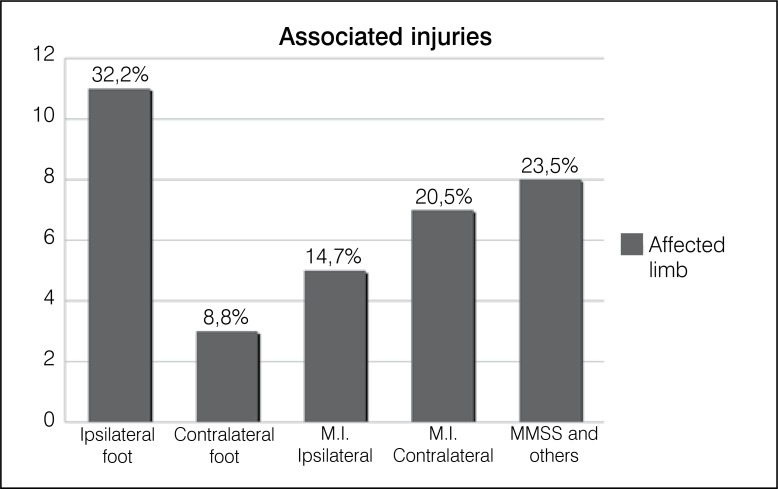
Distribution of cases by associated injuries.

Thirty-four associated injuries of the musculoskeletal system were found, distributed in 22 patients. (Figure 4) The most common site of associated injury was in the ipsilateral foot of the ankle fracture in 11 feet, representing 14.9% of the patients studied.

While most patients used the plaster cast as temporary immobilization device before final fixing, six (8.2%) had definite osteosynthesis performed on the same day of admission, and 18 (24.7%) required reduction of the associated dislocation and installation of a trans-articular external fixation for temporary immobilization of the fracture.

The average time elapsed between the time of the fracture and the definitive healing was 6.5 days, ranging from 0 to 29 days.

In 66 fractures definitive treatment was performed through osteosynthesis, according to the standards recommended by the AO group. Seven cases were treated non- surgically. 

Early complications occurred in 16 cases (21.3%) being infection in 14 cases and wound dehiscence in two cases.

## DISCUSSION

Of our series of 73 patients hospitalized for treatment of malleolar fractures in a tertiary hospital, 63% were male. This finding is in direct contrast with that found by Baptista *et al.* ^11^of only 30.0% of male patients in a study conducted with patients operated at another hospital, with similar characteristics in the same city. As the latter was carried out from 1989 to 1993, we believe that part of this difference can be explained by the time span of 18 years between both studies. However, the predominance of male subjects affected by malleolar fractures has also been found in studies of Santin *et al.* ^12^ and Schwartsmann *et al.* ^14^ with 62.8% and 54.0% respectively. It should be remembered that these two papers have analyzed only Danis-Weber type B malleolarfractures.

It is noteworthy in this study the low mean age of patients, 27.5 years old, with 56.2% of them standing in the age group below 40 years. We believe that the high turnout of traffic accidents, particularly motorcycle accidents, as a cause of fracture contributed to this fact. Debieux *et al*.,^10^ reported a study made in 2001-2002 in which they studied the locomotor injuries due to motorcycle accidents in São Paulo city and found that 79% of patients were under 28 years old. Our finding contrasts with the mean age of 39.0, 48.7, 43.3 and 34.5 years old from the work of Santin *et al*.,^12^ Schwartsmann *et al,* ^14^ Baptista *et al*.^11^ and Tucci Neto* et al*.,^13^ respectively. 

The profile of patients with malleolar fractures treated at a tertiary hospital is well characterized when we analyze trauma mechanisms involved in the genesis of the fracture. We found 54.8% (39 ankles) fractures caused by traffic accidents, and 21.9% (16 ankles) from polytraumatized patients, rates much higher than all other studies in the national literature. Santin *et al*.^12^ described only one fracture due to motorcycle accident (2.9% of the total) and Tucci Neto *et al.* ^13^ had no patient from traffic accident. There is no doubt that Danis-Weber type B fractures evaluated in these two studies are typically caused by torsional trauma, and a lower incidence of fractures due to high-energy trauma was expected. Nevertheless, only 15.4% of patients in the study of Baptista *et al.* ^11^ were due to car accidents, and the same, as already mentioned, was conducted with patients from another major tertiary hospital. 

There is no reference in the work of Baptista *et al*.^11^ of fractures resulting from motorcycle accidents, whereas in our study 26.0% of all fractures resulted from this type of trauma. We believe that the changes that occurred in the transport system of São Paulo city over the 18 years separating the two studies, with the continuous increase in the number of circulating motorcycles, and consequently in the number of accidents involving motorcyclists, are responsible for this change in the etiological pattern. Debieux *et al.* ^10^ showed that only 32 of 387 patients (8.3%), victims of motorcycle accidents used protective boots at the time of the accident. This information is not available for our patients, but since it is a statistical data of this city, it is quite likely that the frequency of use of protective boots is similar. The Brazilian Traffic Code considers a violation only not wearing the helmet, not referring to other protective equipment. The use of boots with high torsional stiffness could prevent ankle fractures. New studies specifically studying malleolar fractures in motorcycle accidents are needed to a better understanding of the injury mechanics in order to support the creation of laws regarding motorcyclists' self-protection equipment. 

The distribution of lesions according to the AO classification, which is the Danis-Weber rating added with subtypes, shows a predominance of type B (56.2%), which is most often studied type of injury in Brazilian literature. We found no epidemiological studies of malleolar fractures that show the relative distribution of the three different types of classification. The finding that 37.0% of fractures of type C is important because it is the fibula fracture with syndesmosis injury, which treatment is more complex than type B. This fact is due to the difficulty in reducing the fibula fracture, particularly in C2 and C3 types, and to the need for perfect restoration of the tíbio-fibular conexion at the syndesmosis level.

It is important to point out the high incidence of fractures in our series. There were 21 cases, 28% of the total, a much higher rate than that found by Baptista *et al*.^11^ of 5.7% and by Santin *et al*.^12^ of 8.8%. Besides the highest index, most of the fractures are of type III according to Gustilo and Anderson classification, nine cases or 42.9%. Of these, five cases were type IIIB, which were ought to be treated by local flaps or microsurgical flaps, through a highly complex procedure only available in few public hospitals. 

Even when it is possible to obtain adequate skin coverage, the prognosis of this type of fracture is usually not good. We have observed that many of these cases progress to a degeneration of the tibiotalar articulation of multifactor etiology, involving cartilage damage at the time of trauma, therefore, the bone destruction that cannot be adequately reconstructed, resulting sometimes in infection and adhesions of periarticular soft tissues. It is quite common that an equinus contracture and a rigid ankle are the end result of a severe open malleolar fracture, regardless how adequate has been the therapeutic conduct, requiring additional procedures as tibiotalar arthrodesis or prosthetic ankle.

Twenty two patients had associated injuries in other parts of the musculoskeletal system (30.1%). In total, 34 different lesions were found, the most frequent injury of the ipsilateral foot of the ankle fracture found in 11 patients, i.e. 50.0% of patients had associated injuries. This should be valued because foot injuries are often underdiagnosed in polytraumatized patients.^3^ ^,^ ^10^ The presence of a fractured ankle on the same side of the foot injury tends to mask the existence of the latter, since the ankle fracture is the most evident injury. As the patient with an ankle fracture cannot walk loading weight to the fractured limb, a possible foot injury may not manifest through pain. Therefore, a careful physical examination looking for traumatic foot injuries should always be performed. Signs such as swelling, bruising and pain on palpation of the foot bones should be valued, since they are all superficial and easily identified. Also keep in mind that ligament injuries of the foot, particularly in the Lisfranc joint are identified on radiographs of the foot only if the radiographic projections are carried out properly and the bone alignment analyzed in detail.

Eighteen of 73 patients (24.7%) studied in this paper presented with associated dislocation of the ankle which required an emergency reduction and installation of an external fixator. This fixator was applied with a modular assembly, two Schanz screws in the tibia and two in the foot, one in the heel and the other at the base of the first metatarsal, a mount that could be changed according to the presence of associated fractures. This procedure aimed to keep the ankle properly reduced in the period preceding the definitive osteosynthesis of fractures, allowing the recovery of soft tissue in both the closed and open fractures. The high average time of 6.5 days between the time of the fracture until definitive treatment is partly explained by the large soft tissue injury in this emergency care. The external fixator as a temporary immobilizer is recently performed procedure in malleolar fractures, and has not been reported in other studies in the Brazilian literature.^11^ ^-^ ^15^ Its use for this purpose is a routine in the management of fractures of the tibial pylon, but with increasing trauma energy in malleolar fractures it has become increasingly common its need in this type of fracture.

The seven patients who were definitively treated non-surgically had this option due to the fact that soft tissue injury did not allow adequate time for internal osteosynthesis. This further point out the high trauma energy involved in the genesis of ankle fractures treated in our department.

We observed 21.3% of acute complications, infection and wound dehiscence, similar to the 25.7% index found by Santin *et al*.^12^ and higher than Baptista *et al*.^11^ (12.7%) and Tucci Neto *et al*.^13^ (4.5%). We attribute this difference to the different characteristics of the fractures treated by us and already discussed earlier in this paper.

## CONCLUSIONS

Ankle fractures treated at a tertiary hospital in a large Brazilian urban center are characterized by: preferentially affects young people around 25 years old; presents as main injury mechanism injury traffic accident; 28% of cases are open fractures (mostly Gustilo and Anderson type C), had high rates of associated injuries (especially in the ipsilateral foot), dislocation is present in 24.7% of cases and evolve with post-operative complications in 21.3% of surgical treatments.
